# Pericranial tenderness in chronic tension-type headache: the Akershus population-based study of chronic headache

**DOI:** 10.1186/1129-2377-15-58

**Published:** 2014-09-05

**Authors:** Kjersti Aaseth, Ragnhild Berling Grande, Christofer Lundqvist, Michael Bjørn Russell

**Affiliations:** 1 Head and Neck Research Group, Research Centre, Akershus University Hospital, 1478 Lørenskog, Norway; 2 Institute of Clinical Medicine, University of Oslo, 1474 Nordbyhagen, Norway; 3 Helse Øst Health Services Research Centre, Akershus University Hospital, 1478 Lørenskog, Norway; 4 Department of Neurology, Akershus University Hospital, 1478 Lørenskog, Norway

**Keywords:** Epidemiology, Population-based, Chronic tension-type headache, Pericranial tenderness

## Abstract

**Background:**

Most knowledge on chronic tension-type headache (CTTH) is based on data from selected clinic populations, while data from the general population is sparse. Since pericranial tenderness is found to be the most prominent finding in CTTH, we wanted to explore the relationship between CTTH and pericranial muscle tenderness in a population-based sample.

**Methods:**

An age- and gender-stratified random sample of 30,000 persons aged 30-44 years from the general population received a mailed questionnaire. Those with a self-reported chronic headache were interviewed and examined by neurological residents. The questionnaire response rate was 71% and the interview participation rate was 74%. The International Classification of Headache Disorders II was used. Pericranial muscle tenderness was assessed by a total tenderness score (TTS) involving 8 pairs of muscles and tendon insertions. Cross-sectional data from the Danish general population using the same scoring system were used for comparison.

**Results:**

The tenderness scores were significantly higher in women than men in all muscle groups. The TTS was significantly higher in those with co-occurrence of migraine compared with those without; 19.3 vs. 16.8, p = 0.02. Those with bilateral CTTH had a significantly higher TTS than those with unilateral CTTH. The TTS decreased significantly with age. People with CTTH had a significantly higher TTS compared to the general population.

**Conclusions:**

People with CTTH have increased pericranial tenderness. Elevated tenderness scores are associated with co-occurrence of migraine, bilateral headache and low age.

Whether the increased muscle tenderness is primary or secondary to the headache should be addressed by future studies.

## Background

Tension-type headache is a common condition throughout the world [1-5]. Pericranial muscle tenderness is found to be the most prominent clinical finding in tension-type headache. It has been postulated that the mechanisms responsible for the increased pericranial tenderness could be peripheral activation or sensitization of myofascial nociceptors. However, evidence for a peripheral abnormality is still lacking [6,7].

Chronic tension-type headache (CTTH) differs from the episodic form in lack of effect of most treatment strategies. CTTH is also associated with overuse of medication and high personal and socioeconomic costs [8]. Prevalence studies report that 3-4% of the adult population has CTTH [2,9]. The pathophysiological mechanisms for CTTH are only partly understood, and it has been debated whether mechanical pain sensitivity is a primary or a secondary phenomenon to CTTH. A 12-year follow-up longitudinal study demonstrated that persons who would later develop CTTH had normal pericranial tenderness scores before the onset of symptoms, which suggests mechanical hypersensitivity to be a consequence rather than a risk factor for the development of CTTH [10]. It has been suggested that pericranial muscle tenderness may not reflect abnormalities within the muscle tissue, but rather sensitization of peripheral nociceptors, second order neurons or a dysfunction in higher order supraspinal pain modulation systems [7]. Other mechanisms also have to be taken into account, since a significant number of people with CTTH in fact do not have increased pericranial muscle tenderness. Several therapeutic approaches have been proposed for the treatment of tension-type headache. However, both behavioral and medical treatment have shown sparse long term effects [11].

Most knowledge is based on data from selected clinic populations, while data from the general population is sparse. The aims of this paper were to describe pericranial muscle tenderness in a large population-based sample of people with CTTH, and explore the correlation of different headache parameters and pericranial muscle tenderness.

## Methods

### Study design and population

This was a cross-sectional population-based study. An age- and gender-stratified sample of 30,000 persons, aged 30–44 years, residing in eastern Akershus County was drawn from the National Personal Registry. Akershus County has both rural and urban areas and is situated in close proximity to Oslo. Data from Statistics Norway show that the sampling area was representative of the total Norwegian population regarding age, gender and marital status. Regarding employment, trade, hotel/restaurant and transport were overrepresented, while industry, oil and gas and financial services were underrepresented in the sampling area compared to the total Norwegian population. The study population received a postal questionnaire. The questions ‘How many days during the past month have you had headache?’ and ‘How many days during the past year have you had headache?’ were used to screen for chronic headache. Those with self-reported chronic headache (i.e. 15 days or more within the past month and/or 180 days or more within the past year) were invited to the Akershus University Hospital. Two neurological residents experienced in headache diagnostics conducted all interviews and the physical and neurological examinations. All headaches were classified according to the explicit diagnostic criteria of the ICHD-II and the revised criteria for medication-overuse headache [12-14]. Patients with CTTH were included into the study, while those with chronic migraine were excluded. The questionnaire response rate was 71%, and the interview participation rate was 74%. Those unable to meet at the clinic were interviewed by telephone. A more detailed description of the materials and methods has been given elsewhere [9,15].

### Pericranial tenderness

A modified version of a previously published pericranial muscle tenderness score system was used to determine the pericranial muscle tenderness [16,17]. Manual pressure was applied to 8 pairs of muscles and/or tendon insertions (m. masseter, m. temporalis, m. frontalis, m. pterygoideus lateralis, m. trapezius, m. sternocleidomastoideus, occipital muscle insertions and mastoid processes). Palpation was made systematically over the surface of the muscle/insertion by applying finger pressure while making small circular movements for 4-5 seconds. The participant’s response was recorded on a 4-point scale as follows: 0 = no visible reaction or verbal report of discomfort, 1 = mild mimic reaction but no verbal report of discomfort, 2 = verbal report and mimic reaction of painful tenderness and discomfort, and 3 = marked grimacing or withdrawal, verbal report of marked painful tenderness and pain. The maximum Total Tenderness Score (TTS) was 48 (8 × 2 × 3 (tender spots × right/left × maximum tender spot score)). The pericranial muscles were divided into two groups, i.e. a cephalic muscle group (frontal, temporal, lateral pterygoid and masseter muscles) and a neck muscle group (insertions at mastoid processes, sternocleidomastoid and trapezius muscles and neck muscle insertions), thus giving a maximum cephalic tenderness score (cephalic TS) of 24 and a maximum neck tenderness score (neck TS) of 24.

Prior to the study, a palpometer, with which a pressure sensitive plastic film attached to the index finger records the pressure exerted, was used to train the observers. No differences in scores were seen between the two observers, which indicates a high inter rater-reliability.

### Reference population

A cross-sectional study of 25–64 year olds from the Danish general population served as reference population [18]. The historical pericranial tenderness scores were compared to our data. The reference population was scored in relation to the splenius and hamulus muscle and coronoid processes, while these tender spots were not included in our study. The reference population was also scored in relation to the anterior and posterior part of the temporal muscle and the profound and superficial part of the masseter muscle, while in our study, there was only one recording for the temporal and masseter muscle. In order to make the scores comparable, the mean value of the two recordings for the temporal and masseter muscles were used, and the reference population tenderness score with 95% confidence intervals was adjusted according to the tender spots investigated in our study.

### Data processing and statistical methods

The statistical analyses were performed using SPSS Base System for Windows 20.0. Different scores are presented as means and 95% confidence intervals. Differences in tenderness scores were assessed with the unpaired Student’s t-test. In the linear regression analysis, migraine and gender were treated as confounders, and the tenderness score was a dependent variable, while age, headache intensity, headache hours per day, headache frequency per month and years with chronic headache were treated as predictor variables.

### Ethical issues

The Regional Committees for Medical Research Ethics and the Norwegian Social Science Data Services approved the project. Participation was based on signed informed consent.

## Results

Of the 386 participants with chronic headache, 28 had chronic migraine and 358 had CTTH. Two hundred ninety-nine participants (71 men and 228 women), were examined for pericranial muscle tenderness and eligible for this study, while the 87 participants exclusively interviewed by telephone were excluded.

We found no significant differences in total tenderness score (TTS) between those with chronic migraine and CTTH (21.4 vs. 19.7, p = 0.5). Two hundred seventy-five participants (64 men and 211 women) were diagnosed with CTTH with or without medication overuse.

Table 1 shows the distribution of co-occurrence of migraine and medication overuse. The TTS was significantly higher in those with than without co-occurrence of migraine (19.3 vs. 16.8, p = 0.02 (men 14.7 vs. 11.5, p = 0.2; women 20.1 vs. 19.0, p = 0.4)). Similarly, neck tenderness score (neck TS) was significantly higher in those with migraine (13.2 vs. 11.2, p = 0.03) while no significant differences were found in cephalic tenderness score (cephalic TS).

**Table 1:** Distribution of migraine and medication overuse in chronic tension-type headache

		**Migraine (n)**		
		**+**	-	**Total**
Medication overuse (n)	+	59	56	133
	-	58	102	166
	Total	117	158	275

We found no significant differences in TTS between those with and without medication overuse (17.6 vs. 18.0, p = 0.8 (men 11.7 vs. 12.9, p = 0.5; women 19.6 vs. 19.4, p = 0.9)). Neck TS and cephalic TS were equal in those with and without medication overuse (for cephalic TS 5.6 vs 6.1, p = 0.4 and for neck TS 12.1 vs. 12.0, p = 0.9).

The different subgroups of medication overuse (i.e. overuse of paracetamole N = 68, NSAIDs N = 47, combination analgesics N = 26 and triptans N = 1) were similar in TTS, but those overusing NSAIDs tended to have a slightly lower TTS than those overusing paracetamole (both gender 16.8 vs. 20.0, p = 0.1,( men 10.6 vs.13.4, p = 0.3, and women 19.5 vs. 21.9, p = 0.3). Co-occurrence of migraine did not affect the results.

**Figure 1:**
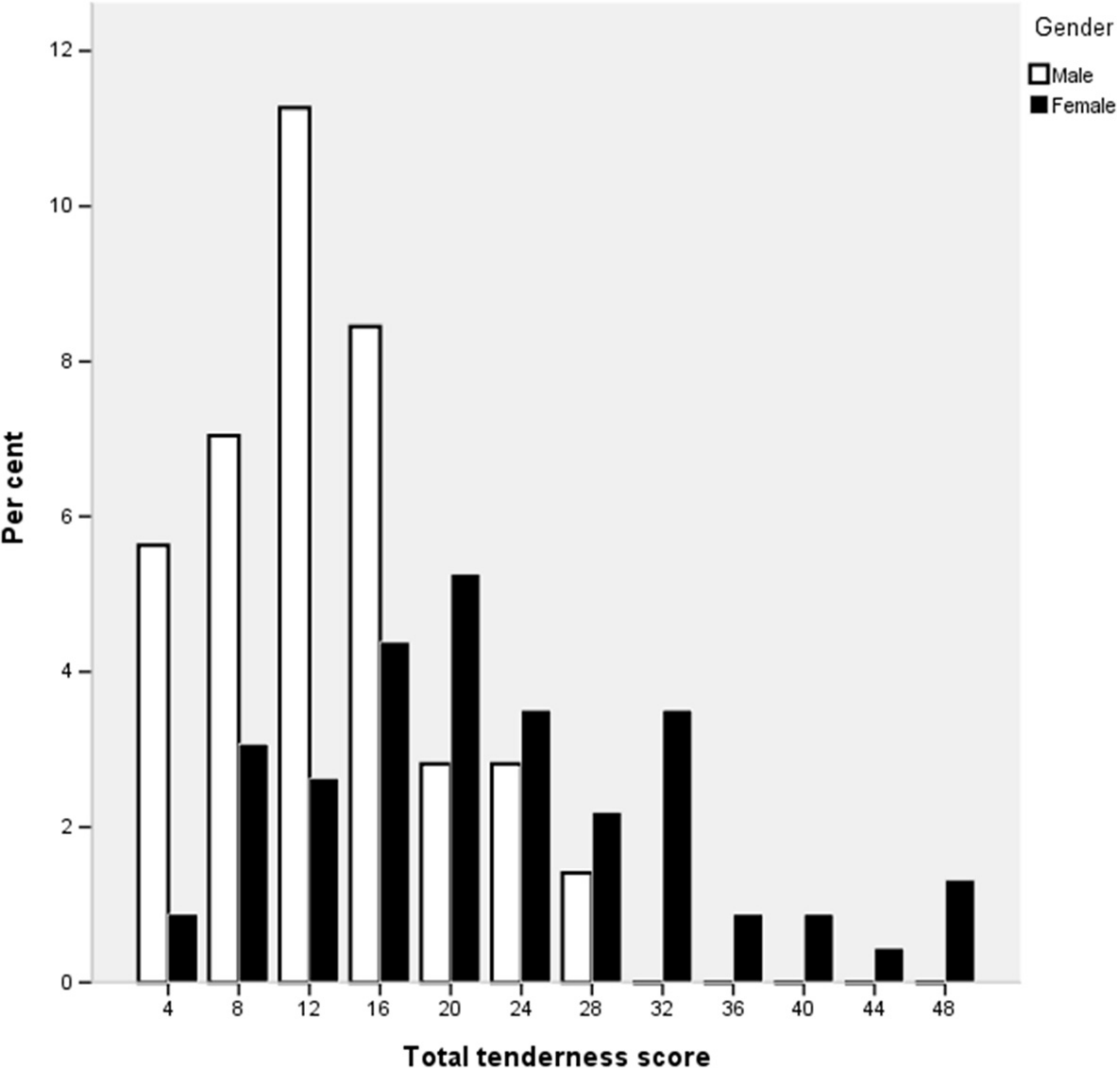
Total tenderness scores by gender in people with chronic tension-type headache.

Figure 1 shows the distribution of the TTS. Women were significantly more tender than men in all examined muscles. Table 2 shows that the neck TS were higher than the cephalic TS. Those with CTTH were significantly more tender than people from the general population. Table 3 shows that those with bilateral CTTH had a significantly higher TTS than those with unilateral CTTH. The same tendency was found in cephalic TS and neck TS. Those with unilateral CTTH had a tendency for a slightly higher TTS on the headache than non-headache side.

**Table 2:** Tenderness scores in people with chronic tension-type headache (CTTH)

**Muscle/insertion**	**Mean total tenderness score (95% CI)**	
	**Males (N = 64)**	**Females (N = 211)**
M. frontalis	0.5 (0.3-0.8)	0.9 (0.8-1.1)
M. temporalis	0.6 (0.4-0.9)	1.3 (1.1-1.5)
M masseter	1.4 (0.8-2.0)	2.1 (1.9-2.3)
M. pterygoideus lateralis	1.0 (0.6-1.3)	2.2 (2.0-2.5)
Processus mastoideus	1.1 (0.8-1.4)	2.1 (1.9-2.3)
M. sternocleidomastoideus	2.8 (2.4-3.3)	3.4 (3.2-3.7)
M. trapezius	2.4 (2.0-2.7)	3.7 (3.2-4.1)
Neck muscle insertion	2.7 (2.2-3.1)	3.8 (3.6-4.1)
Cephalic tenderness score	3.5 (2.5-4.4)	6.5 (5.9-7.2)
Neck tenderness score	8.9 (7.6-10.1)	13.0 (12.3-13.7)
Total dxt. tenderness score	6.1 (5.2-6.9)	9.9 (9.2-10.5)
Total sin. tenderness score	6.3 (5.3-7.3)	9.7 (9.0-10.3)
Total tenderness score CTTH population	12.3 (10.5-14.1)	19.5 (18.3-20.7)
Total tenderness score general population*	7 (6-8)	14 (13-15)

*Jensen et al. Muscle tenderness and pressure pain thresholds in headache. A population study. Pain 1993;52:193-9.

CI denotes confidence interval.

**Table 3:** Tenderness Scores (TS) by gender in people with bilateral and unilateral chronic tension-type headache (CTTH)

**Gender**	**Localization**	**n**	**Right TS**	**Left TS**	Cephalic TS*	Neck TS**	**Total TS**
	**CTTH**						
Both	Bilateral	248	9.2 (8.6-9.8)	9.0 (8.5-9.7)	6.0 (5.4-6.6)	12.3 (11.6-12.9)	18.3 (17.3-19.2)
	Unilateral	27	6.8 (5.2-8.4)	7.3 (5.4-9.1)	4.4 (2.9-5.9)	9.6 (7.5-11.8)	14.0 (10.7-17.3)
	*Right*	12	6.6 (4.4-8.7)	5.9 (3.5-8.4)	3.1 (1.2-4.9)	9.4 (6.4-12.5)	12.5 (8.6-16.3)
	*Left*	15	6.9 (4.3-9.6)	8.3 (5.3-11.4)	5.5 (2.9-8.0)	9.8 (6.3-13.3)	15.3 (9.7-20.8)
Male	Bilateral	56	6.3 (5.4-7.2)	6.6 (5.5-7.7)	3.5 (2.4-4.6)	9.4 (8.0-10.7)	12.9 (10.9-14.9)
	Unilateral	8	4.4 (2.9-5.9)	4.1 (2.8-5.4)	3.1 (1.5-4.8)	5.4 (3.3-7.5)	8.5 (6.2-10.9)
	*Right*	3	5.0 (3.1-6.9)	3.3 (1.6-5.1)	2.3 (-1.4-5.1)	6.0 (2.7-9.3)	8.3 (5.1-11.6)
	*Left*	5	4.0 (1.8-6.2)	4.6 (2.7-6.5)	3.6 (0.4-6.8)	5.0 (0.7-9.3)	8.6 (5.0-12.2)
Female	Bilateral	192	10.1 (9.5-10.7)	9.8 (9.1-10.4)	6.7 (6.0-7.4)	13.1 (12.4-13.9)	19.8 (18.7-21.1)
	Unilateral	19	7.8 (5.6-10.0)	8.6 (5.9-11.2)	5.0 (2.7-7.1)	11.4 (8.6-14.3)	16.4 (11.6-21.2)
	*Right*	9	7.1 (4.2-10.0)	6.8 (3.6-10.0)	3.3 (0.5-6.1)	10.6 (6.0-15.1)	13.9 (7.9-19.9)
	*Left*	10	8.4 (5.3-11.5)	10.2 (6.7-13.7)	6.4 (2.7-10.1)	12.2 (7.8-16.7)	18.6 (12.1-25.1)

*In cephalic TS frontal, temporal, lateral pterygoid and masseter muscles are included.

**In neck TS insertions at mastoid processes, sternocleidomastoid and trapezius muscles and neck muscle insertions are included.

The brackets denote 95% confidence intervals.

Table 4 shows that TTS and neck TS decreased significantly with age. Cephalic TS was significantly correlated with headache intensity. None of the other outcome variables headache hours per day, headache frequency per month or years with chronic headache were correlated with TTS, cephalic TS or neck TS. However, the TTS tended to increase with increasing headache intensity.

**Table 4:** Correlations between headache clinical parameters and total tenderness score (TTS), cephalic and neck tenderness score in multiple regression

**Variable**	**Total TS**		**Cephalic TS**		**Neck TS**	
	**Regr. coeff**	**p-value**	**Regr. coeff**	**p-value**	**Regr. coeff**	**p-value**
Age	−0.40	0.003	−0.06	0.41	−0.34	0.000
Duration (hours)	0.03	0.87	0.04	0.63	−0.02	0.87
Frequency (days)	0.16	0.16	0.05	0.38	0.10	0.11
Headache years	0.02	0.82	0.01	0.78	0.01	0.90
Intensity (VAS)	0.52	0.06	0.32	0.04	0.20	0.21

## Discussion

The purpose of the present study was to describe pericranial muscle tenderness in a population based sample of people with CTTH. Quantification of pericranial muscle tenderness in a population-based sample of CTTH has, to our knowledge, not been studied earlier.

Our main findings indicate that increased pericranial tenderness is associated with co-occurrence of migraine, bilateral headache and young age. The pericranial tenderness was significantly elevated in those with CTTH compared to the general population. Headache intensity, headache hours per day, headache frequency per month and years with chronic headache did not seem to have influence on pericranial tenderness.

Previous studies have shown conflicting results of pericranial muscle tenderness in migraine, i.e. increased muscle tenderness during attacks and in headache-free periods [19,20], while other authors have found the pericranial tenderness alone to be closely related to frequency of coexisting tension-type headache [18]. One study identified increased pericranial tenderness on the symptomatic side compared to the non-symptomatic side in patients with strictly unilateral migraine [21]. We have earlier demonstrated that migraine and tension-type headache are strongly interrelated [22]. It has been postulated that the mechanisms responsible for the increased pericranial tenderness could be peripheral activation or sensitization of myofascial nociceptors. Thus, the elevated tenderness score might be explained by central sensitization and might in turn be involved in the generation of chronified headache. Migraine has previously been shown to be associated with central sensitization [23,24], which may conceivably explain our higher tenderness scores in those with co-occurrence of migraine.

We found that those with unilateral CTTH had a significantly lower TTS when compared to those with bilateral CTTH. They did not have co-occurrence of migraine more frequently. The pathophysiological mechanisms responsible for unilateral CTTH are not known. However, we cannot exclude the possibility of more focal sensitization in the activated unilateral pain signaling pathways. Such mechanisms may be peripheral or central. People with cervicogenic headache have significantly higher pericranial muscle tenderness score on the pain than non-pain side, suggesting local factors in the neck playing a role [25]. Our CTTH sample included too few persons with unilateral headache to draw firm conclusions. We found, however, a tendency towards higher muscle tenderness on the headache-side than the non-headache side, which is in accordance with findings in cervicogenic headache.

One population-based study has found association between pericranial muscle tenderness and headache frequency in tension-type headache [18]. In the referred study, episodic and chronic tension-type headache were compared, while in our study, all participants were diagnosed with CTTH (i.e. >15 days per month). We found no significant associations between pericranial muscle tenderness and headache pain intensity, headache hours per day, headache frequency per month and years with chronic headache, which is in accordance with a study on college students with CTTH [26], and a recent study of a clinic based sample of CTTH sufferers [6].

We found that women obtained a higher TTS than men, and that the tenderness decreased with increasing age, confirming a previous study from the general population where the same tendency was found in the general population [27]. A considerable number of people with CTTH were without increased pericranial tenderness. Thus, the origin of the pain cannot be explained by local muscular factors, and supports the view that other pathophysiological mechanisms also are involved.

### Methodological considerations

Our large population-based sample with a high participation rate provides data representative of the general population. The sample size was chosen to ensure adequate numbers of people with chronic headache for accurate descriptive statistics. The age range of 30–44 years was chosen because the prevalence of chronic headache is higher in this group than in younger people, whereas co morbidity of other diseases is lower than in older age groups. The recipients of our questionnaire were informed that we conducted an investigation about headache, but they did not receive specific details about our focus in order to minimize selection bias. The ICHD-II classification was used for headache diagnoses. The general aspects, limitations and strengths of this study have been discussed in details elsewhere [9,15].

We used a population-based reference population for comparison of pericranial muscle tenderness. The reference population was representative for the Danish and the Norwegian general populations regarding age, gender and marital status. The Danish reference population had a wider age range than our sample, and the data was besides collected 15 years earlier. In the Danish study, the invitation was framed as an offer of a thorough health examination and the importance of participation of all subjects invited was emphasized. In neither of the studies they were informed of the hypotheses of the study.

## Conclusions

People with CTTH have significantly increased pericranial tenderness compared with the general population. We found co-occurrence of migraine, bilateral headache and low age to be associated with increased pericranial tenderness in CTTH. The different pathophysiological mechanisms of the tenderness and its role in pain sensitization in recurrent or chronic headache disorders are not fully understood. Whether mechanical pain sensitivity is a primary or a secondary phenomenon to recurrent headache is still under debate and further studies are needed.

## Competing interests

The authors declare that they have no competing interests.

## Authors’ contributions

MBR had the original idea for the study and planned the overall design. KA prepared the initial draft and was the main author of the present manuscript. RBG and KA collected data. CL and MBR was involved in data analysis and interpretation and assisted in preparation of the manuscript. All authors read and approved the manuscript.
